# Effect of partially hydrolyzed synbiotic formula milk on weight gain of late preterm and term infants—a multicenter study

**DOI:** 10.3389/fped.2023.1270442

**Published:** 2023-10-20

**Authors:** Suzan Sahin, Mehmet Buyuktiryaki, Nilufer Okur, Abdullah Baris Akcan, Mehmet Fatih Deveci, Sadik Yurttutan, Sezgin Gunes, Ayse Anik, Ramazan Ozdemir, Ozgun Uygur, Mehmet Yekta Oncel

**Affiliations:** ^1^ Faculty of Medicine, Department of Pediatrics, Division of Neonatology, Izmir Democracy University, Izmir, Türkiye; ^2^ Faculty of Medicine, Department of Pediatrics, Division of Neonatology, Istanbul Medipol University, Istanbul, Türkiye; ^3^ Department of Pediatrics, Division of Neonatology, Gazi Yasargil Training and Research Hospital, Diyarbakir, Türkiye; ^4^ Faculty of Medicine, Department of Pediatrics, Division of Neonatology, Adnan Menderes University, Aydin, Türkiye; ^5^ Faculty of Medicine, Turgut Ozal Medical Center, Department of Pediatrics, Division of Neonatology, Inonu University, Malatya, Türkiye; ^6^ Faculty of Medicine, Department of Pediatrics, Division of Neonatology, Kahramanmaras Sutcu Imam University, Kahramanmaras, Türkiye; ^7^ Department of Pediatrics, Division of Neonatology, Buca Seyfi Demirsoy Training and Research Hospital, Izmir, Türkiye; ^8^ Department of Pediatrics, Division of Neonatology, Tepecik Training and Research Hospital, Izmir Health Sciences University, Izmir, Türkiye; ^9^ Faculty of Medicine, Department of Pediatrics, Division of Neonatology, Izmir Katip Celebi University, Izmir, Türkiye

**Keywords:** synbiotic formula, partially hydrolyzed formula, infant growth, formula tolerance, side effect

## Abstract

**Introduction:**

Data on the effectiveness of hydrolyzed infant formula containing both pre- and probiotics (synbiotic formula) on the growth of infants is still scarce. This retrospective study was designed to evaluate the effect of a partially hydrolyzed synbiotic formula on growth parameters and the possible occurrence of major gastrointestinal adverse events or morbidities in infants born via cesarean section (C-section) delivery.

**Methods:**

C-section-delivered term and late preterm infants who received either partially hydrolyzed synbiotic formula, standard formula, or maternal milk and followed at seven different hospitals from five different regions of Turkey, during a 1-year period with a minimum follow-up duration of 3 months were evaluated retrospectively. All the included infants were evaluated for their growth patterns and any kind of morbidity such as diarrhea, constipation, vomiting, infection, or history of hospitalization.

**Results:**

A total of 198 infants (73 in the human milk group, 61 in the standard formula group, and 64 in the partially hydrolyzed synbiotic formula group) reached the final analysis. The groups were similar regarding their demographic and perinatal characteristics. No difference was observed between the three groups regarding gastrointestinal major side effects. Growth velocities of the infants in the human milk and partially hydrolyzed synbiotic formula groups during the first month of life were similar whereas the weight gain of infants in the standard formula group was significantly less than these two groups (*p *< 0.001). Growth velocities were similar among the three groups between 1st and 3rd months of age.

**Discussion:**

A partially hydrolyzed synbiotic formula provided better weight gain in late-preterm and term infants who were delivered via C-section delivery compared to the standard formula during the first month of life. This weight gain was similar to the infants receiving exclusively human milk. This difference was not observed in length and head circumference gain. No difference was observed in any of the parameters during the 1st–3rd months of age. Specially formulated partially hydrolyzed synbiotic formulas may reverse at least some of the negative impacts of C-section delivery on the infant and help to provide better growth, especially during the early periods of life.

## Introduction

Several constituents found in human breast milk play vital roles in facilitating early human growth and development (1, 2). Early human survival relies on this source of nutrition, which is irreplaceable (2–4). From the very first minutes of life, it furnishes the infant with not only essential nutrients but also probiotic and prebiotic support (5). Thus, to foster healthy microbiota in infants, exclusive breastfeeding is very important.

When certain mothers cannot breastfeed their infants, it becomes necessary to use infant formulas that imitate human milk. Subsequently, each formula's safety and effectiveness ought to undergo testing (6).

Human beings are inhabited by specialized microbiota that have coevolved functions, actively contributing to human health, development, and resistance to diseases. Healthy microbiota have a significant impact on the growth and development of infants (7).

Balanced and diverse microbiota help in the efficient absorption of nutrients from breast milk or formula. This aids in providing the necessary energy and building blocks for healthy growth (2).

*Bifidobacterium* and *Bacteroides* species are known to be maternally transmitted to infants mostly through vaginal delivery (7). However, infants born by cesarean section (C-section) have been observed to have a predominance of *Enterobacteriaceae* family members, such as *Klebsiella* and *Enterobacter* in their gut. The influence of this fact on the host's health remains largely unexplored (7–9). Whether there is a direct causal relationship with the microbiota or not, epidemiological studies have revealed correlations between C-section delivery and immune disorders as well as metabolic disorders (10–12).

Despite all attempts, C-section delivery rates are unfortunately still high in parts of the world, especially in our country (13). While the effects of C-section delivery are clear, little is known about the impact of nutrition on infants born by C-section. Both breastfeeding and vaginal delivery positively affect the formation of an infant's intestinal microbiota and protect the infant from many diseases (14). Therefore, adding prebiotics to infant formulas along with probiotics is the new trend of constantly evolving technology in infant nutrition. Chin et al. have previously shown that the negative effect of C-section on microbiota balance can be reversed with synbiotic-containing formula (formula containing both pre- and probiotics) support (15).

In addition to the results of many studies investigating the effects of microbiota regulation on allergy, immunity, and inflammation, in the study conducted by Wang et al., it was observed that daily weight gain similar to breastfed infants can be achieved with synbiotic-containing formula (16, 17).

It is still a matter of curiosity how these infants will grow compared to standard formula or breast-fed infants. It is not clear whether they can match breast milk-fed infants' growth, or whether side effects of the formula such as constipation will develop. In very rare studies, extensively hydrolyzed formula containing B. breve M-16 V and the prebiotic blend scGOS/lcFOS (9:1) has been shown to promote adequate infant growth (1, 16).

However, data on the effectiveness of hydrolyzed synbiotic formulas on growth of infants is still scarce. The present retrospective study was designed to evaluate the effect of a partially hydrolyzed synbiotic formula on the daily weight gain of infants born via C-section delivery along with their weekly length and head circumference gain and development of major gastrointestinal adverse events or morbidities. Our hypothesis in this study was that synbiotic formulas may provide better growth in C-section-delivered infants compared to standard formulas.

## Methods

This retrospective multicenter study was conducted in seven different hospitals from five different regions of Turkey, over a 1-year period. Ethical approval was obtained for the study from the local ethics committee. Apart from this approval, local ethical approval from each center was also obtained.

All the term and late preterm infants who were followed at outpatient clinics of Istanbul Medipol University, Gazi Yasargil Training and Research Hospital, Inonu University, Adnan Menderes University, Izmir Buca Seyfi Demirsoy Training and Research Hospital, Tepecik Training and Research Hospital, and Kahramanmaras Sutcu Imam University were assessed for eligibility. Infants who were born via vaginal delivery or not meeting inclusion criteria such as receiving mixed feeding (infants who received both maternal milk and formula milk) from the beginning of their lives were excluded at the first step. Other exclusion criteria were history of hospitalization in NICU, having congenital anomaly, being SGA (small for gestational age) or LGA (large for gestational age), insufficiency of the data in the medical records, and infants from whom parental consent could not be obtained.

**Figure 1:**
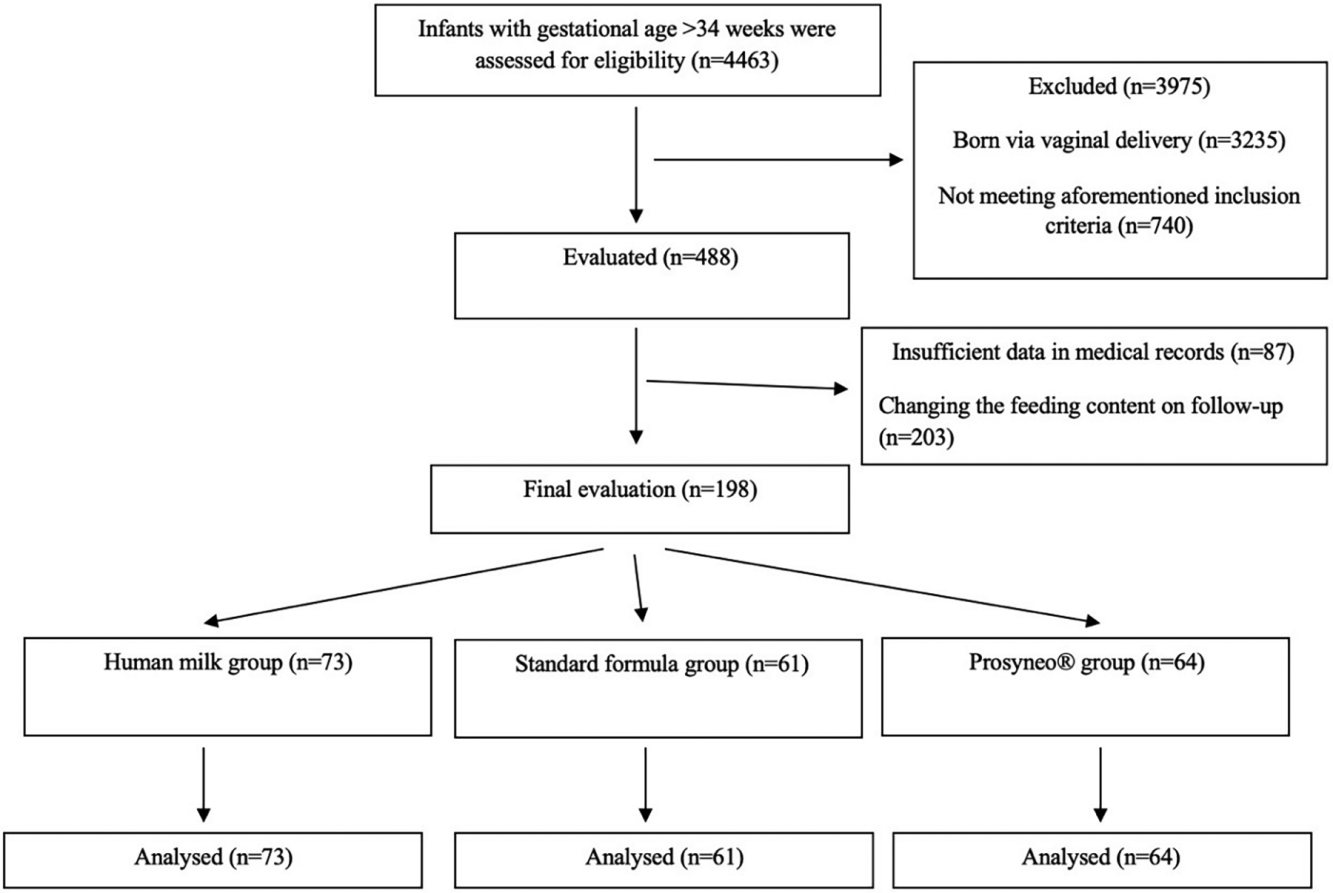
Flowchart of study.

The follow-up of all the infants had been made by attending neonatologists from each center. Follow-up data were also recorded and evaluated retrospectively. The included infants were already 3 months old. They were separated into three different categories according to the content of their feeding. One group had been exclusively breastfeeding and one group received standard nonhydrolyzed cow's milk formula and the other received partially hydrolyzed synbiotic formula containing scGOS/lcFOS (9:1) and B. breve M-16 V (Prosyneo®). The infants who had insufficient data in their medical records or received another type of feeding content were also excluded before the final evaluation. Finally, all the included infants had already completed their routine 3-month follow-up control visits. The flow diagram and number in each group according to this classification are shown in Figure 1. All the infants in the standard formula group had received the same standard formula. The contents of the two different formulas are shown in Table 1.

**Table 1:** Macronutrient contents of the two different formulas in the study.

	Standard formula	Prosyneo®	Unit
Energy	67	65	kcal/100 ml
Fat	3.5	3.4	g/100 ml
Carbohydrate	7.6	7.2	g/100 ml
Fiber	0.8	0.8	g/100 ml
Protein	1.4	1.5	g/100 ml

Apart from the demographic characteristics, all the included infants were evaluated for their growth patterns and any kind of morbidity such as diarrhea, constipation, vomiting, infection, or history of hospitalization. Medically confirmed adverse events that had been indicated throughout the investigated period were also recorded. All of the measurements were performed by the same attending neonatologists from each center. In order to weigh an infant, a thin piece of soft paper was placed on the scale. Then, the fully naked infant was placed on the scale and the neonatologist waited until the infant was stable. Finally, the infant's weight was recorded to the nearest 0.1 kg. The length of the infant was measured by two healthcare providers. The infant was laid face-up on an infantometer. One of the healthcare providers positioned the head gently against the headplate as the other straightened the legs. The footplate was pressed gently to the bottom of the feet. The measurement on the infantometer was recorded as the length of the infant. In order to measure the head circumference of the infant, a measuring tape that cannot be stretched was used. It is wrapped tightly around the widest circumference of the head, starting from the most prominent part of the forehead across to the most prominent part of the occipital bone and then to the forehead again. This measurement was repeated three times in order to minimize measuring errors and the largest number was recorded.

Diarrhea was diagnosed if stools suddenly increased in number and looseness in 3 or more stools and/or contained mucus, blood, or a bad smell. Constipation was defined as when an infant had bowel movements and defecation less frequently than they used to and/or painful defecation and/or hard or pellet-like stools. Vomiting was defined as pathologic when it was in large amounts, projectile, bilious or there were signs of illness and feeding refusal. Data regarding these aforementioned parameters were obtained from the medical charts of the infants which were recorded during their routine outpatient follow-up visits. Follow-up charts for to the first 3 months of their life were evaluated.

### Primary and secondary outcomes

The primary outcome of this study was daily weight gain and weekly increase in length and head circumference of included infants who had been fed with the aforementioned different types of feeding content. The secondary outcome was the development of major gastrointestinal adverse events or morbidities in these infants.

### Statistical analysis

The data were analyzed using IBM SPSS Statistics (Windows, Version 20.0. Armonk, NY: IBM Corp.). Descriptive statistics were used including mean [with standard deviations (SD)] and median (min-max) for continuous variables, and counts (proportions) for categorical variables. Student's *t*-test and the Mann–Whitney *U*-test compared continuous variables for parametric and non-parametric variables, respectively. X2 or Fischer's exact test was used to analyze categorical variables. The difference among the three groups was examined using a repeated measures ANOVA test. Statistical significance was considered if the *p*-value was <0.05.

## Results

A total of 4,463 infants followed-up in five different out-patient clinics with gestational ages of >34 weeks were assessed for eligibility. In total, 3,235 infants were excluded as they had been delivered via vaginal delivery. Furthermore, 740 infants were excluded due to the aforementioned exclusion criteria and 290 more infants were excluded due to insufficiency of data in medical records (such as absent values for at least one of the anthropometric measures, not attending their follow-up visits, etc.) (*n* = 87, 17.8%) or due to receiving another type of feeding content due to family's choice or changing conditions, on follow-up (*n* = 203, 41.6%). The percentage of these losses due to feeding content changes was similar in the three groups. Ultimately, a total of 198 infants, 73 in the human milk group, 61 in the standard formula group, and 64 in the partially hydrolyzed synbiotic formula group reached final analysis (Figure 1).

The demographics and perinatal characteristics of study infants were similar between the three groups (Table 2). The mean gestational ages of the infants were 38.1 ± 1.2, 38 ± 1.1, and 38.2 ± 1.4 weeks and mean birth weights were 3,223 ± 526, 3,201 ± 416, and 3,161 ± 432 respectively for the human milk group, standard formula, and partially hydrolyzed synbiotic formula groups (*p* = 0.805; *p* = 0.617). All the infants were singletons. When the infants were compared regarding gastrointestinal major adverse events or morbidities such as recurrent vomiting, intractable diarrhea, constipation, significant flatulence, bloody stools, necrotizing enterocolitis, or increased number of infantile colic episodes attributed to the content of feeding, there was a weaker tendency in the infants receiving the partially hydrolyzed synbiotic formula compared to standard formula-fed infants even if this did not reach statistical significance (4.7% in symbiotic formula group vs. 6.6% in standard formula group) (Table 3).

**Table 2:** Demographics and perinatal characteristics.

	Human milk group (*n* = 73)	Standard formula group (*n* = 61)	Prosyneo® group (*n* = 64)	*p*-value
Gestational age, week	38.1 ± 1.2	38 ± 1.1	38.2 ± 1.4	0.805
Birth weight, g	3,223 ± 526	3,201 ± 416	3,161 ± 432	0.617
Birth length, cm	49.5 ± 2.4	49.2 ± 2.1	49.6 ± 1.8	0.626
Birth head circumference, cm	34.4 ± 1.5	34.4 ± 1.2	34.6 ± 1.1	0.735
Maternal age, years	28 ± 6	30 ± 6	28 ± 5	0.092
Gender, male, *n* (%)	36 (49.3)	33 (54.1)	34 (53.1)	0.839
1 min. APGAR median (min-max)	8 (6–9)	8 (6–9)	8 (4–9)	0.304
5 min. APGAR median (min-max)	9 (8–10)	9 (8–10)	9 (7–10)	0.262
Consanguinity, *n* (%)	9 (12.3)	4 (6.6)	3 (4.7)	0.516
Gestational diabetes mellitus, *n* (%)	4 (5.5)	6 (9.8)	5 (7.8)	0.635
Atopy history, *n* (%)	4 (5.5%)	3 (4.9%)	1 (1.6%)	0.467

Data presented as mean ± SD, median (min-max) or count (percentages).

**Table 3:** Comparison of gastrointestinal major adverse events and morbidities attributed to the content of feeding.

	Human milk group (*n* = 73)	Standard formula group (*n* = 61)	Prosyneo® group (*n* = 64)	*p*-value
Adverse event/morbidity^a^, *n* (%)	2 (2.7)	4 (6.6)	3 (4.7)	0.300
Vomiting, *n* (%)	1 (1.4)	1 (1.6)	1 (1.6)	0.991
Diarrhea, *n* (%)	0 (0)	0 (0)	1 (1.6)	0.349
Constipation, *n* (%)	1 (1.4)	3 (4.9)	1 (1.6)	0.358

^a^ Data presented as count (percentage).

The mean weight/length/head circumference of infants in the 1st month of life was 4,358 ± 615 gr/53 ± 3/36.9 ± 1.7 cm in the human milk group, 3,977 ± 633 gr/53 ± 2/36.7 ± 1.3 cm in the standard formula group, and 4,068 ± 627 gr/53 ± 2/37 ± 1.3 cm in the partially hydrolyzed synbiotic formula group. The mean weight/length/head circumference of infants in the 3rd month of life was 6,158 ± 733 gr/60 ± 3/40.1 ± 1.5 cm in the human milk group, 5,890 ± 590 gr/59 ± 2/40.3 ± 1.4 cm in the standard formula group, and 6,027 ± 780 gr/60 ± 3/40.1 ± 1.5 cm in the partially hydrolyzed synbiotic formula group. The growth velocities of the infants are shown in Table 4. During the first month (Period 1), the weight gain of infants in the standard formula group was significantly less than the other two groups while the infants in the partially hydrolyzed synbiotic formula group gained weight similar to the human milk group (*p* < 0.001). When the weight gain during Period 2 was compared, no statistical significance was observed in any of the groups. Length and head circumference gains during both the Period 1 and 2 were similar between the three groups (Table 4). None of the infants had serious infections with the need for hospital outpatient clinic admission, treatment, and/or hospitalization.

**Table 4:** Growth velocities of the infants.

	Human milk group (*n* = 73)	Standard formula group (*n* = 61)	Prosyneo® group (*n* = 64)	*p*-value
Weight gain till 1st month, g/day	48 ± 18	35 ± 25	46 ± 16	**<0**.**001**
Weight gain between 1st and 3rd months, g/day	30 ± 10	32 ± 8	33 ± 8	0.153
Length gain till 1st month, mean ± SD, cm/week	0.90 ± 0.48	0.86 ± 0.45	0.96 ± 0.42	0.263
Length gain between 1st and 3rd months, cm/week	0.78 ± 0.25	0.78 ± 0.26	0.85 ± 0.23	0.170
Head circumference gain till 1st month, mean ± SD, cm/week	0.62 ± 0.34	0.60 ± 0.28	0.70 ± 0.31	0.113
Head circumference gain between 1st and 3rd months, cm/week	0.39 ± 0.16	0.44 ± 0.14	0.39 ± 0.16	0.255

Data presented as mean ± SD.

Statistically significant *p*-value is shown bold.

## Discussion

Whether partially hydrolyzed synbiotic infant formula is superior to standard formula in infant growth is still uncertain. In this retrospective study, we aimed to investigate the difference in growth parameters in C-section-delivered late preterm and term infants according to the types of their feeding content.

In a randomized controlled double-blind prospective multicenter study comparing a similar extensively hydrolyzed synbiotic formula with the same formula without a synbiotic content, average weight gain (g/day) during the study period was similar between the synbiotic and control groups. This result is different than our results specifically for the first month of life (18). Our results regarding the partially hydrolyzed synbiotic formula revealed weight gain during the first month of life similar to human milk and better than standard formula. This means that when weight gain is the issue, this formula could provide results similar to maternal milk. Furthermore, in the results of our study, it was shown to be not inferior to and even better than the standard formula.

Recently, in a prospective randomized double-blind controlled trial, conducted in term healthy Chinese infants, it was shown that a similar partially hydrolyzed synbiotic infant formula when compared to nonhydrolyzed prebiotic formula without probiotics ensured adequate growth and was well tolerated (16). Similarly, we observed that the partially hydrolyzed synbiotic formula had been well tolerated by all of the study infants without statistically significant adverse events.

It has been shown that delayed settlement of *bifidobacteria* persisted until 2–3 months of life in infants after being delivered by C-section and early supplementation of pre- and probiotics provided fast restoration of the intestinal colonization (15). This settlement is a keystone contributing to immune modulation and maintaining a favorable environment in the infant's intestine which will help tolerance and minimize adverse events (17, 19–22). Previous other studies have also shown that synbiotic formulas with similar content were well tolerated (15, 18, 23, 24). Thus, with our study, literature support on the use of the partially hydrolyzed synbiotic infant formula has been strengthened by a retrospectively designed study.

In our study, even if the weekly length gains of infants during both of the time periods in the partially hydrolyzed synbiotic infant formula group were more than the other two groups, this did not reach statistical significance. Abrahamse-Berkeveld et al. did not observe significant differences in length or head circumference gain between the standard or synbiotic formula-fed infant groups (18). According to the current literature, it could be expected that infants fed with formulas would have a higher length and/or weight gain compared to infants on human milk (25). This may be attributed to the small sample size and/or limited time period evaluated in our study. Along with this, it is a fact that formula-fed infants are the ones with insufficient maternal milk supply. Like many countries, the healthcare providers in our country recommend the least amount of formula to be given to the infant in order to foster maternal lactation (26). Thus, as long as the infants are not overfed, they may be expected to achieve similar growth patterns both in length and weight in the long term period. Consistent with this knowledge, growth in all three parameters was found to be similar in all three groups during the second time period (1st–3rd months). Thus, this special formula also ensured adequate weight, length, and head circumference gain throughout the investigated period similar to the maternal milk.

There are some limitations in this study. First of all, this study has a retrospective design. This design did not enable us to make a detailed analysis of the fecal content. Thus, the results of the current study are just a glimpse into the possible outcomes of further prospective and well-structured studies. We do not have exact data regarding the total daily intakes of each infant, which is also a limitation. Another issue is that long-term results for the anthropometric measures and growth velocities, possible allergies, infection frequency, neurodevelopmental alterations, and incidence of metabolic syndrome would give more valuable information about the effect of the partially hydrolyzed synbiotic infant formula. It is known that formula-fed infants typically gain weight more quickly after about 2–3 months of age (27, 28). A study investigating growth velocities for a longer period would give more precise results. As these kinds of special formulas usually cost more than standard formulas, this fact may also be considered in further studies and cost-benefit analyses should be considered. Last but not least, not only differences in weight gain but also body composition should be evaluated for more precise comparisons (6). Thus, we highly recommend investigators perform further studies evaluating body composition changes over time as well. This article is one of the very rare studies investigating the effects of a partially hydrolyzed synbiotic formula on infant growth parameters and possible adverse events that the infant may experience during their follow-up period. As this is one of the first studies published on this subject, we hope to make a valuable contribution to the current literature.

Finally, as the second step of the study, the 18–24 month-old infants are planned to be evaluated for their growth parameters, neurodevelopmental outcomes, and the presence of any kind of allergy diagnosis in order to present more valuable data to the literature.

## Conclusion

A partially hydrolyzed synbiotic formula could provide better weight gain in late-preterm and term infants who were delivered via C-section delivery compared to the standard formula during the first month of life. This weight gain was similar to the infants receiving exclusively human milk. This difference was not observed in length and head circumference gain. No difference was observed in any of the parameters during the 1st–3rd months of age. Gastrointestinal system-related morbidities were slightly less in partially hydrolyzed synbiotic formula-receiving infants compared to standard formula-fed infants even if this did not reach statistical significance. Specially formulated partially hydrolyzed synbiotic formulas may reverse at least some of the negative impacts of C-section delivery on the infant and help to provide better growth, especially during the early periods of life. The reasons underlying this effect may be investigated in further larger and prospective studies.

## Data Availability

The original contributions presented in the study are included in the article/Supplementary Material, further inquiries can be directed to the corresponding author.
